# Common trunk branching from the renal artery to the diaphragm, adrenal gland, and testis: a case report with embryological hypotheses by observation

**DOI:** 10.1007/s12565-025-00829-2

**Published:** 2025-03-07

**Authors:** Yoko Ueda, Wataru Nemoto, Rio Hosoda, Hayato Terayama, Kenta Nagahori, Daisuke Kiyoshima, Zhe-Wu Jin, Takashi Okazaki, Masahito Yamamoto, Kaori Suyama, Shogo Hayashi

**Affiliations:** 1 Department of Anatomy, Division of Basic Medical Science, Tokai University School of Medicine, 143, Shimokasuya, Isehara-shi, Kanagawa 259-1193 Japan; 2 Tokai University School of Medicine, 143, Shimokasuya, Isehara-shi, Kanagawa 259-1193 Japan; 3 Department of Anatomy, Wuxi School of Medicine, Jiangnan University, No. 1800, Lihu Avenue, Wuxi, 214122 Jiangsu China; 4 Department of Radiology, Tokai University School of Medicine, 143, Shimokasuya, Isehara-shi, Kanagawa 259-1193 Japan

**Keywords:** Adrenal glands/blood supply, Anatomic variation, Diaphragm/blood supply, Renal artery, Testis/blood supply

## Abstract

The vascular systems of the kidneys and gonads are highly susceptible to variations because of the degeneration of the mesonephros and mesonephric artery and the progressive ascending development of the metanephros. In this report, we present a rare case of a common arterial trunk originating from the left renal artery and supplying the left diaphragm, adrenal gland, and testis. Anatomical dissection of a 95-year-old male was conducted during the 2023 academic year at Tokai University Medical School. The left renal artery had a common trunk that bifurcated to superior and inferior branches, forming a T-shape. The superior branch was distributed in the left adrenal gland and diaphragm, whereas the inferior branch was distributed in the left testis. This anatomical variation likely results from the remnant of the mesonephric artery network structure, particularly the rete arteriosum urogenitale. Understanding such variations is crucial for radiologists and surgeons to avoid potential complications during diagnostic procedures and surgical interventions.

## Introduction

The arteries branching from the abdominal aorta into the kidneys, diaphragm, adrenal glands, and gonads generally include the inferior phrenic, adrenal, renal, and gonadal arteries. Among these, the inferior phrenic artery is a pair of arteries that originate from the abdominal aorta or celiac trunk and are distributed in the diaphragm. The three types of adrenal arteries are the superior adrenal artery, arising from the inferior phrenic artery; the middle adrenal artery, arising from the abdominal aorta; and the inferior adrenal artery, arising from the renal artery. The gonadal arteries (testicular or ovarian) typically arise from the abdominal aorta inferior to the renal arteries and descend to supply the respective gonads (Wacker et al. 2017). The renal veins drain into the inferior vena cava. The right gonadal and adrenal veins drain directly into the inferior vena cava, whereas the left gonadal and adrenal veins drain into the left renal vein.

The aforementioned vascular systems of the kidneys, adrenal glands, and gonads are highly susceptible to variation. Numerous studies have reported on the origin, distribution, and course of the renal artery and its branches (Wacker et al. 2017). Herein, we describe a case of a common trunk branching to the inferior adrenal artery, testicular arteries that did not branch from the normal position (abdominal aorta), and a branch that distributed in the diaphragm. We further discuss the potential embryological basis for this unique variation and its clinical importance, considering the development of the renal vasculature.

## Case report

During routine anatomical dissection of a 95-year-old male cadaver (cause of death, hyperglycemic, hyperosmolar syndrome) at Tokai University in 2023 after 10% formalin perfusion via the femoral artery, a unique vascular variation was observed on the left side. The authors hereby confirm that every effort was made to comply with all local and international ethical guidelines and laws concerning the use of human cadaveric donors in anatomical research.

The cadaver was donated via a donor-based system (Tokai Daigaku Kentai No Kai) following the guidelines included in the Act on Body Donation for Medical and Dental Education (Body Donation Law) in Japan. Ethical committee permission was not required because the individual donated his body for anatomical research and education. This study complied with the guidelines of the Japanese Association of Anatomists. Informed consent was obtained from the individual ante-mortem person through Tokai Daigaku Kentai No Kai.

**Fig. 1:**
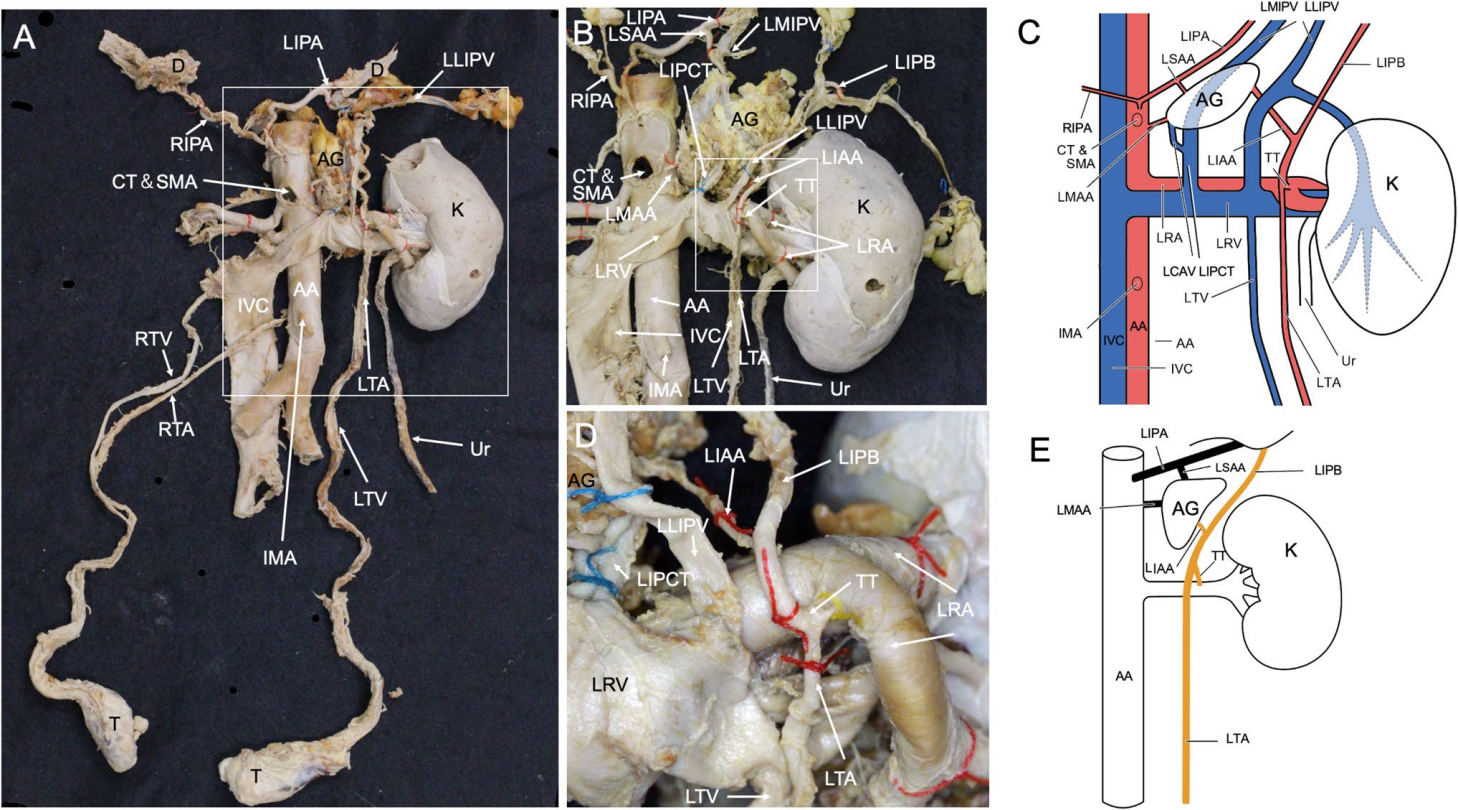
Kidney and the surrounding vasculature observed in this case. **A** Overview. **B** Enlarged view of the kidney and abdominal aorta area enclosed in a white box of **A**. **C** Schematic diagram of the kidney and surrounding vasculature in **B**. **D** Enlarged view of the renal artery, T-shaped trunk, inferior adrenal artery, inferior phrenic branch, and testicular artery enclosed in a white box of **B**, **E** simplified schematic diagram of the aorta, showing the testicular and inferior phrenic arteries branching off from the renal artery in a T-shaped common trunk. AA, abdominal aorta; AG, adrenal gland; CT, celiac trunk; D, diaphragm; IMA, inferior mesenteric artery; IVC, inferior vena cava; K, kidney; LAV, left adrenal vein; LCAV, left central adrenal vein; LIAA, left inferior adrenal artery; LIPA, left inferior phrenic artery; LIPB, left inferior phrenic branch; LIPCT, left inferior phrenic common trunk; LMIPV, left medial inferior phrenic vein; LLIPV, left lateral inferior phrenic vein; LMAA, left middle adrenal artery; LRA, left renal artery; LRV, left renal vein; LSAA, left superior adrenal artery; LTA, left testicular artery; LTV, left testicular vein; RIPA, right inferior phrenic artery; RTA, right testicular artery; RTV, right testicular vein; SMA, superior mesenteric artery; T, testis; TT, T-shaped common trunk; Ur, ureter

The left renal artery bifurcated into two branches immediately before entering the hilum into the anterior and posterior sides of the left renal vein (Fig. 1A,B,C,D). Before this bifurcation, one artery branched off from the renal artery in an anterior upper direction, forming a common trunk that bifurcated to superior (cranial) and inferior (caudal) branches, forming a T-shape (Fig. 1B, C,D,E). The superior branch then further divided into two branches, one to the diaphragm and the other to the adrenal glands. We define the one that distributed to the diaphragm as the inferior phrenic branch and the other that distributed to the adrenal glands as the inferior adrenal artery. The inferior phrenic branch was distributed toward the underside of the diaphragm. The inferior adrenal branch originated from the superior branch of the common trunk. The inferior branch spread to the left testis and functioned as the testicular artery.

On the right side, the right kidney had two renal arteries originating from the abdominal aorta at the level of the second lumbar vertebra. However, the abdominal aorta showed variation, with the celiac trunk and superior mesenteric artery originating from the common conduit.

The left renal vein receives the left testicular and left adrenal veins, which drain into the inferior vena cava. In this case, there were two left inferior phrenic veins: the left medial inferior phrenic vein and the left lateral inferior phrenic vein. A small vein drained from the dorsal side of the left adrenal gland and flowed into the medial inferior phrenic vein passing through the dorsal side. In addition, the left central adrenal vein draining from inside the adrenal gland joined the left inferior phrenic vein at a left inferior phrenic common trunk and flowed into the left renal vein. In this way, the left adrenal vein merged with a branch of the left lateral inferior phrenic vein. In the current case, the thin left lateral inferior phrenic vein was distributed in the diaphragm, renal fascia (Gerota’s fascia), and perirenal fat that converged into a thick vein and also drained into the left renal vein (Fig. 1A, B, C). The right and left kidneys exhibited normal morphology.

## Discussion

**Fig. 2:**
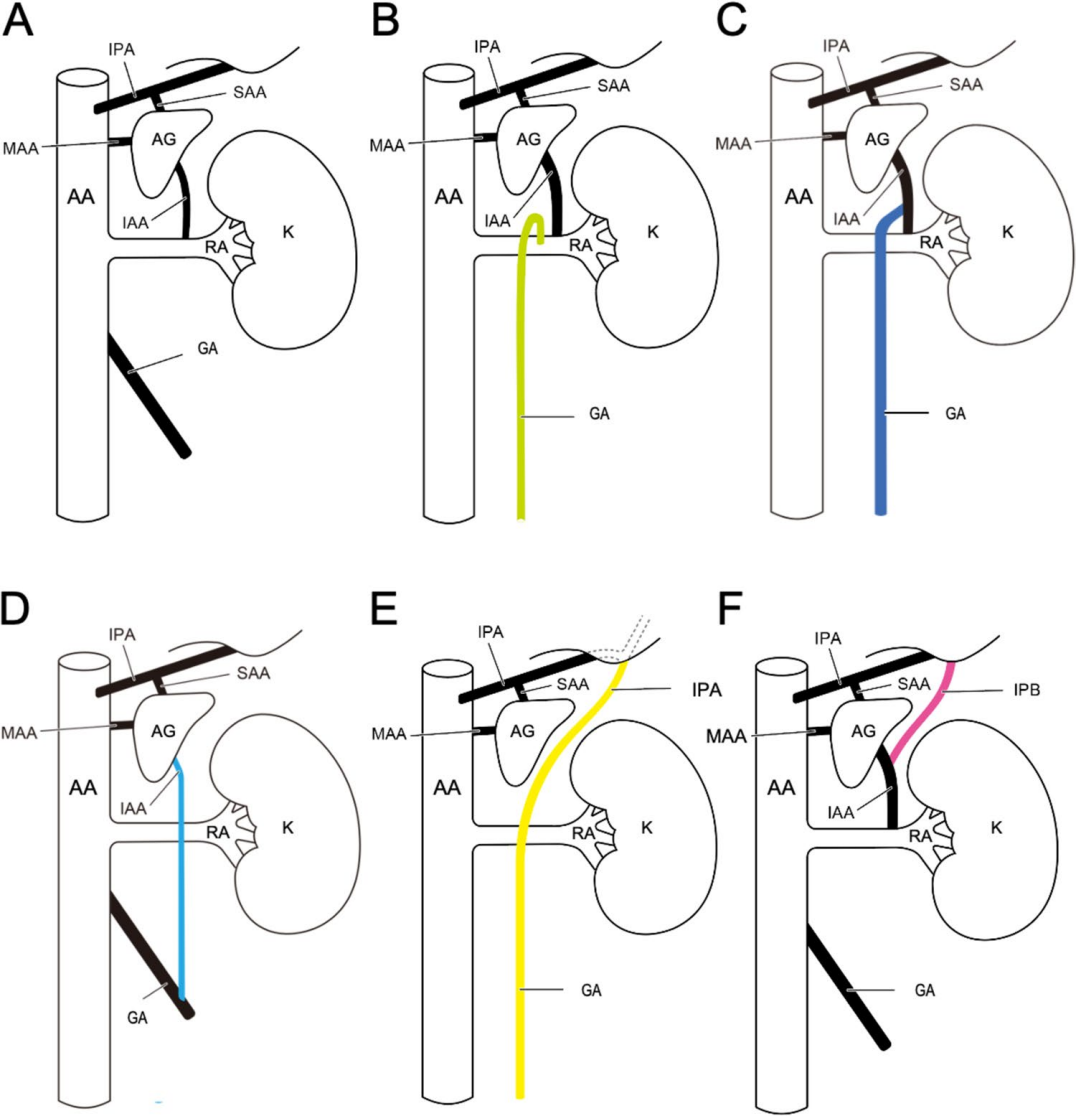
Schematic diagram of the variations reported in previous studies that are related to the present case. **A** Normal morphology. **B** GA displacement from AA to RA. **C** GA displacement from AA to IAA. **D** IAA displacement from RA to GA. **E** GA displacement from AA to IPA. **F** IPB (a branch of the IPA) to IAA. AA, abdominal aorta; AG, adrenal gland; GA, gonadal artery (ovarian or testicular arteries); IAA, inferior adrenal artery; IPA, inferior phrenic artery; IPB, inferior phrenic branch; K, kidney; MAA, middle adrenal artery; RA, renal artery; SAA, superior adrenal artery

Typical vascular branching of the kidney and the surrounding adrenal gland, diaphragm, and gonad of adults are shown in Fig. 2A. A few cases of arterial branching, isolated variations of the inferior adrenal, inferior phrenic, and testicular arteries are commonly described in the literature. They are partially related to that observed in our case. Based on the displacement of the testicular artery from the abdominal aorta to renal artery, the right testicular artery branching from the abdominal aorta and the left testicular artery branching from the left renal artery occur in 4% of cases (Wacker et al. 2017). Branching of the testicular artery in the anterosuperior surface of the renal artery, which is rare (percentage unknown; Fig. 2B; Petru et al. 2007). A mutation that resulted in the testicular artery being displaced from the abdominal aorta to the inferior adrenal artery (percentage unknown; Fig. 2C; Inoue and Masuko 1981). Displacement of the inferior adrenal artery from the renal artery to become a branch of the testicular artery is extremely rare (percentage unknown; Fig. 2D; Bordei et al. 2003; Shanthakumar et al. 2016). A case in which the inferior adrenal artery was absent and the right gonadal artery branched off from the inferior phrenic artery (0.12%) was reported by Khamanarong et al. (2012). Meanwhile, Merklin and Michels (1958) reported that the gonadal artery rarely (percentage unknown) arises from the inferior phrenic artery (Fig. 2E).

Displacement of the inferior adrenal artery and the inferior phrenic branch from the abdominal aorta to the inferior phrenic artery has been reported. In such cases, the inferior phrenic branch is defined as a branch distributed in the diaphragm in addition to the inferior phrenic artery (Koizumi 2007). The inferior phrenic branch originating from the inferior adrenal artery is observed in 17.5% (Fig. 2F; Bordei et al. 2003). To the best of our knowledge, a common trunk arising from the renal artery and giving rise to the inferior adrenal artery, inferior phrenic branch, and testicular artery (as a ventral T-shaped branch) has not been previously reported.

The embryological development of these vessels can provide insights into the origin of this anomaly. The inferior phrenic, adrenal, and gonadal arteries may originate from the arterial network supplying the mesonephros (Topaz et al. 2010). During fetal development, the function of the mesonephros is excretion; however, it disappears by the 8th week of gestation. The metanephros, which is newly formed caudally, ascends to form the definitive kidney. Generally, the ladder theory of metanephric ascension by Felix’s (1912) is used to explain the renal vascular system. Felix (1912) noted that the mesonephric arteries branching from the aorta and extending laterally to the mesonephros are distributed in a ladder-like fashion, suggesting that as the metanephros ascends, parts of this ladder remain and become the inferior adrenal, inferior phrenic, and renal arteries and part of the gonadal artery. The network theory, proposed by Evans (1909), posits that renal arteries develop from the capillary network surrounding the mesonephros. Hochstetter (1893) reported that the ascending metanephros incorporates vessels from the branches of the dorsal aorta, which ultimately become the final renal arteries. Isogai et al. (2010) reported that the vascular system of the mesonephros disappears completely along with it, and the vessels necessary for the ascending metanephros are newly formed directly from the abdominal aorta.

We propose that the rete arteriosum urogenitale, as described by Felix (1912), provides a potential explanation for the common trunk and its branches observed in the present case. The rete arteriosum urogenitale corresponds to persistent branches of anomalous arterial supply to the kidneys (Kónya 1985). Even after testicular descent, the arteries originally supplying the mesonephros and gonad primordia likely retain some of their original distribution, contributing to the formation of the rete arteriosum urogenitale (Mine and Shimada 2002). In our case, the craniocaudal orientation of the common trunk and its bifurcation to the testicular artery, inferior adrenal artery, and inferior phrenic branch further suggests a close relationship with the craniocaudally oriented rete arteriosum urogenitale. Moreover, we hypothesize that the dorsal and ventral vascular networks of the rete arteriosum may explain both the dorsal branching pattern of the testicular artery from the renal artery, as previously reported (Fig. 2B; Petru et al. 2007), and the dorsal orientation of the T-shaped common trunk observed in our case.

Clinically, variations such as those observed in the present case require caution during vascular imaging examinations such as magnetic resonance imaging and computed tomography as well as during surgery involving the retroperitoneal organs such as the kidneys and adrenal glands. Variations in the branches of the renal artery are important in sympathectomies performed for patients with hypertension (renal denervation; Sato et al. 2021), pose risks in kidney transplantation if multiple branches are present (Budhiraja et al. 2010), and can affect the success rate of embolization procedures for cancer treatment (White et al. 2021). Keeping the embryological origins of the vessels in mind can help predict variations and avoid clinical complications.

## Data Availability

No datasets were generated or analyzed during the study.

## References

[CR1] Bordei P, St Antohe DS, Sapte E, Iliescu D (2003) Morphological aspects of the inferior suprarenal artery. Surg Radiol Anat 25:247–251 14504822 10.1007/s00276-003-0132-z

[CR2] Budhiraja V, Rastogi R, Asthana AK (2010) Renal artery variations: embryological basis and surgical correlation. Rom J Morphol Embryol 51:533–536 20809032

[CR3] Evans HM (1909) On the development of the aortae, cardinal and umbilical veins, and the other blood vessels of vertebrate embryos from capillaries. Anat Rec 3:498–518

[CR4] Felix W (1912) Mesonephric arteries (aa. Mesonephricae). In: Keibel F, Mall FP (eds) Manual of human embryology. Lippincott, Philadelphia, pp 820–825

[CR5] Hochstetter F (1893) Beitrage zur entwicklungsgeschichte des venen-systems der amnioten: III [Contribution to the developmental history of the vein system of amniotes: III]. Sauger Morph Jahrb 20:542–542

[CR6] Inoue K, Masuko S (1981) A case of testicular arteries from the inferior suprarenal arteries of both sides. Chiba Med J 57:209–211

[CR7] Isogai S, Horiguchi M, Hitomi J (2010) The para-aortic ridge plays a key role in the formation of the renal, adrenal and gonadal vascular systems. J Anat 216:656–670 20579173 10.1111/j.1469-7580.2010.01230.x PMC2952379

[CR8] Khamanarong K, Woraputtaporn W, Amarttayakong P et al (2012) The right ovarian artery arising from the right inferior phrenic artery: a case report. J Med Assoc Thai 95:743–745 22994039

[CR9] Koizumi M (2007) Gross anatomy of the inferior phrenic artery and the collateral pathways to the diaphragm. Jpn Respir Soc Clin Anat 7:52–53

[CR10] Kónya A (1985) An unusual congenital abnormality of the renal artery: left renal artery arising from the right renal artery. Br J Radiol 58:891–893 3842292 10.1259/0007-1285-58-693-891

[CR11] Merklin RJ, Michels NA (1958) The variant renal and suprarenal blood supply with data on the inferior phrenic, ureteral and gonadal arteries: a statistical analysis based on 185 dissections and review of the literature. J Int Coll Surg 29:41–76 13502578

[CR12] Mine K, Shimada K (2002) Superior testicular branch arising from the epididymal artery. Jpn Res Soc Clin Anat 3:14–15

[CR13] Petru B, Elena S, Dan I, Constantin D (2007) The morphology and the surgical importance of the gonadal arteries originating from the renal artery. Surg Radiol Anat 29:367–371 17593308 10.1007/s00276-007-0224-2

[CR14] Sato Y, Kawakami R, Jinnouchi H et al (2021) Comprehensive assessment of human accessory renal artery periarterial renal sympathetic nerve distribution. JACC Cardiovasc Interv 14:304–315 33541541 10.1016/j.jcin.2020.09.043

[CR15] Shanthakumar RS, Kumar N, Badagabettu SN, Kappettu Gadahad MRK, Reghunathan D, Patil J (2016) Rare combined variation of left suprarenal vessels associated with retroaortic left renal vein. Proc Singap Healthc 25:112–114

[CR16] Topaz O, Topaz A, Polkampally PR, Damiano T, King CA (2010) Origin of a common trunk for the inferior phrenic arteries from the right renal artery: a new anatomic vascular variant with clinical implications. Cardiovasc Revasc Med 11:57–62 20129362 10.1016/j.carrev.2009.09.002

[CR17] Wacker F, Lippert H, Pabst R (2017) Arterial variations in humans: key reference for radiologists and surgeons. Classification and frequency. Thieme Medical Publishers, New York

[CR18] White RD, Moore KS, Salahia MG et al (2021) Renal arteries revisited: anatomy, pathologic entities, and implications for endovascular management. Radiographics 41:909–928 33939544 10.1148/rg.2021200162

